# Comparative analysis of meat quality of Laiwu black, Minxinan black and Hyla rabbits

**DOI:** 10.5194/aab-67-503-2024

**Published:** 2024-10-08

**Authors:** Beibei Zhou, Liping Yang, Yajia Zhang, Xianfeng Yan, Haitao Sun, Ce Liu, Yin Zhang, Liya Bai, Haihua Zhang

**Affiliations:** 1 Key Laboratory of Livestock and Poultry Multi-omics of MARA, Institute of Animal Science and Veterinary Medicine, Shandong Academy of Agricultural Sciences, Jinan 250100, China; 2 Hebei Key Laboratory of Specialty Animal Germplasm Resources Exploration and Innovation, College of Animal Science and Technology, Hebei Normal University of Science and Technology, Qinhuangdao 066000, China; 3 Shandong Animal Husbandry Station, Jinan 250100, China

## Abstract

The meat rabbit industry in China relies on foreign breeds and synthetic lines; the development of superior domestic breeds has not yet been fully realised. We compared the meat quality of two Chinese local breeds of black rabbits (Laiwu black and Minxinan black) and Hyla commercial rabbits, to provide a reference for the utilisation of meat rabbit breeds. In the present study, 35 d old Laiwu black rabbits, Minxinan black rabbits, and Hyla rabbits (20 rabbits each) were selected and reared under identical feeding and management conditions for 7 weeks, after which 10 randomly selected rabbits from each group were slaughtered for the analysis of meat quality. The $a^{*}$ (redness) value of the two local breeds was significantly higher than that of Hyla rabbits ( p<0.01 ). The shearing force and drip loss of Laiwu black rabbits were significantly lower than those of the other groups ( p<0.05 ). The two local breeds exhibited significantly higher myoglobin levels than Hyla rabbits ( p<0.01 ), while melanin was highest in the meat of Minxinan black rabbits. The essential amino acids (valine, isoleucine, leucine and lysine) were significantly lower in Minxinan black rabbits than in the other groups ( p<0.05 ). Aldehyde (heptanal, octanal) contents in Minxinan black meat were significantly higher than those of the other groups ( p<0.01 ); however, nucleotide (guanine, adenine) contents were significantly lower ( p<0.01 ). Unsaturated fatty acids (oleic, linoleic, α -linolenic) were significantly higher in the meat of Laiwu black rabbits than in those of the other groups ( p<0.05 ). Both Laiwu black and Minxinan black rabbits have certain advantages in terms of nutritional indicators while also having good meat colour and flavour. The results of this study provide a theoretical reference for the research and development of meat rabbit breeds.

## 1 Introduction

Rabbit meat is a highly digestible, tasty, low-calorie food that has recognised nutritional properties. In particular, it is lower in fat (5.3 % vs. 9 % in chicken) and cholesterol than other types of meat and is rich in protein (Jiang et al., 2020). Rabbit meat is popular in European countries, America, Japan and other countries, where it is considered to be a healthy meat option. China is a major producer of rabbit meat, and domestic consumption of rabbit meat is also increasing. The rabbit meat production of China occupies a decisive position in the world. Since 2000, the rabbit meat production of China has always been maintained at over 40 %, accounting for over 50 % of the global rabbit meat production and thereby becoming the largest producer of rabbit meat compared to other countries. However, the Chinese meat rabbit industry relies heavily on foreign breeds and synthetic lines, with the development of superior domestic breeds having not yet been fully realised (C. Liu et al., 2021). Laiwu black and Minxinan black rabbits are local black meat rabbit breeds that are used in China. They have been included in the National Breed List of Livestock and Poultry Genetic Resources. The Laiwu black rabbits primarily originate from the Laiwu district of Shandong Province, which is located in eastern China. Laiwu black rabbits belong to medium local breeds with black fur and exhibit strong fecundity, rapid growth and superior meat quality (Zhou et al., 2023). The Minxinan black rabbit, a small local breed with black fur in Fujian Province, which is in the south of China, demonstrates robust resilience to adversity, wide adaptability and improved meat quality (Chen et al., 2023). Minxinan black rabbits were introduced in Shandong Province by our research team in 2018 for domestication and breeding purposes. Hyla, a foreign introduced synthetic line rabbit with white fur, currently has the largest market share in Chinese domestic meat rabbit industry. Previous studies have shown that the meat quality and organoleptic property of local black rabbits are better than those of imported white rabbits with similar body weight (Wang et al., 2016; Liu et al., 2022).

Research shows that the meat quality and nutritional value of black-coat livestock is higher than that of white-coat livestock. Consumers also prefer to purchase black livestock and poultry, increasing their economic values. Kadaknath chicken meat is rich in bioactive dipeptide carnosine and is a nutritional source of anserine and creatinine (Sharma et al., 2022). Black-bonded chickens have been considered to be a highly nutritious food with high meat quality, specifically in terms of good taste, juiciness, high melanin content, and richness in polyunsaturated fatty acids and flavour amino acids (Dou et al., 2022). Black pork has a pleasant meat colour and marbling, high protein content, and high intramuscular fat content. Experimental studies have shown that Berkshire pork is a superior nutritional source of monounsaturated fatty acids, vitamins and minerals (Subramaniyan et al., 2016). A survey showed that Croatian consumers preferred black pigs over meat from hybrid pigs (Jelić Milković et al., 2023). It has been reported that the fresh-tasting amino acid content is higher and bitter-tasting amino acids are lower in Jeju black-cattle beef than in Hanwoo beef (Lee et al., 2019). Although black goat meat has a unique odour, it is used as a nutritional food for human consumption because of its low fat, low cholesterol and high protein contents (Choi et al., 2023).

However, there is limited research that has been conducted on the quality and nutritional value of black-rabbit meat. In this study, in order to explore differences in the meat quality between local black and imported white rabbits, Laiwu black rabbits, Minxinan black rabbits and Hyla commercial rabbits were selected for meat quality testing. The results of this study provide a theoretical reference for the research and development of rabbit meat breeds.

## 2 Material and methods

### 2.1 Experimental design

Each group had 20 rabbits (equally split between male and female), consisting of 35 d-old Laiwu black rabbits, Minxinan black rabbits and Hyla commercial rabbits, respectively. There was a total of 60 rabbits spread over three groups. Within each group, rabbits had similar body weights, came from maternal rabbits with live-litter sizes of seven to eight, and were not siblings or half-siblings. Two rabbits in each group were raised in a commercial cage ( 60cm×41cm×35cm ). All rabbits were housed in a closed and ventilated house where the temperature range was 20–25 °C and where the relative humidity range was 50 %–60 %. During the experiment, rabbits were fed ad libitum and drank freely. The material composition and nutrient level of the basic diet were in accordance with the details shown in Table 1 (Zhou et al., 2023). After 7 weeks, 10 randomly selected rabbits (equally split between male and female) from each group were slaughtered for the analysis of meat quality. The rabbits were slaughtered (according to the standard guidelines for euthanising rabbits) at 84 d of age after 24 h of fasting. This experiment was carried out under Laiwu Black Rabbit Seed Industry Technology Co., Ltd., Jinan, Shandong, China.

**Table 1:** Composition and nutrient levels of diets (air-dry basis).

Ingredients	Content	Nutrient levels ${}^{b}$	Content
Corn	25.0	Digestible energy /( $\mathrm{MJ}\;{\mathrm{kg}}^{-1}$ )	9.90
Soybean meal	17.0	Crude protein	16.26
Wheat bran	11.0	Neutral-detergent fibre	31.27
Wheat middling	10.0	Acid detergent fibre	20.39
Alfalfa meal	8.0	Acid detergent lignin	6.26
Peanut vine	8.0	Crude fat	2.55
Peanut shell	19.0	Calcium	0.84
Calcium hydrogen phosphate	0.5	Total phosphorus	0.44
Calcium carbonate	0.5	Lysine	0.79
Premix ${}^{a}$	1.0	Methionine and cysteine	0.56
Total	100.0		

${}^{a}$ The premix provided the following per-kilogram diet: vitamin A – 10 000 IU, vitamin D3 – 1500 IU, vitamin E – 50 mg, vitamin K3 – 3.0 mg, thiamine 5.0 mg – riboflavin – 10 mg, pantothenic acid – 20 mg, nicotinic acid – 50 mg, Fe – 100 mg, Zn – 30 mg, Cu – 20 mg, Mn – 30 mg, choline – 400 mg, NaCl – 5.0 g, lysine – 1.0 g, methionine – 1.0 g. The rest is a miscellaneous meal carrier complement. ${}^{b}$ Digestible energy was a calculated value, while the others were measured values.

### 2.2 Slaughter performance

The pre-slaughter, half-bore and full-bore weights were measured. The half-bore and full-bore rates were then calculated. Half-bore weight was defined as the carcass weight excluding the blood, fur, head, tail, limbs (the forelimbs at the wrist and the hindlimbs at the hock), intestine and contents, urinary and reproductive organs, trachea, esophagus, and gallbladder. Full-bore weight was defined as the half-bore weight excluding heart, liver, kidney and perirenal fat. We took 8 samples (equally split between male and female) of leg muscles and 10 samples (equally split between male and female) of two longissimus dorsal from each group. We then selected, from the eight samples of leg muscle from each group, one male and one female meat sample to mix into one portion stored at 4 °C for subsequent experiments. The 10 samples (equally split between male and female) of two longissimus dorsal from each group were also placed in a cold storage chamber at a temperature of 4 °C for subsequent experiments.

1Half-bore rate(%)=(half-bore weight/pre-slaughter weight)×100.2Full-bore rate(%)=(full-bore weight/pre-slaughter weight)×100.

### 2.3 Meat quality

The pH of the meat was measured at 45 min and 24 h post-slaughter using a pH meter (PHBJ-206, China). The probe of the PH meter was inserted directly into the muscle to a depth of 3 mm. Objective colours, including $a^{*}$ (redness), $b^{*}$ (yellowness) and $L^{*}$ (lightness), were measured 45 min post-slaughter using a colorimeter (NR20XE, 3nh, China) by means of reflectance spectroscopy on the fresh cross-section of the longissimus muscle. The difference in the weight of the collected and stored samples ( <45 min post-slaughter and >24 h storage in a refrigerator at 4 °C ) was determined.

Drip loss was calculated as the percentage of the weight difference. The longissimus dorsal was trimmed into a suitable size within 2 h after slaughter and was weighted for an initial weight, followed by placement in a muscle drip loss measurement tube at 4 °C for 24 h. Then, the sample was taken out, and filter paper was used to absorb the surface moisture of the meat sample, which was then weighed for a final weight. The standard formula is as follows:

3 $$\begin{array}{rl} & \text{drip loss}\;(\%)=\\ & [(\text{initial weight}-\text{final weight})/\text{initial weight}]\times 100. \end{array}$$

The shearing force, water loss and cooking loss were measured 48 h post-slaughter. The shearing force was measured using a meat tenderness meter (C-LM3, USA). Water loss was determined using the filter paper press method. Cooking loss was measured using the weight change percentage (Daszkiewicz and Gugołek, 2020).

### 2.4 Nutrient components

The contents (%) of moisture, protein, lipid and ash in meat were analysed according to the method of Y. Zhang et al. (2022) with some modifications. The contents (%) of vitamin E and nicotinic acid were analysed using high-performance liquid chromatography according to the guidelines of the GB5009.82–2016 National Food Safety standard determination of vitamins A, D and E in foods and the GB5009.5–2016 National Food Safety standard determination of niacin and nicotinamide in foods.

The composition and percentage content (%) of amino acids were analysed by means of a high-speed amino acid analyser (Hitachi, LA8080). Then 0.4 g of the sample was weighed, hydrolysed and filtered. The processed samples were placed in 50 mL volumetric flasks; subsequently, 1.0 mL of the sample was transferred to a 15 mL test tube with reduced pressure to dry at 40 °C and then was dissolved with 1.0 mL of sodium citrate buffer solution with pH=2.2 and measured on the machine through membrane filtration.

### 2.5 Melanin and myoglobin

#### 2.5.1 Melanin content

The melanin content in the meat was determined using an enzyme-linked immunosorbent assay (ELISA) kit (Jiangsu Meimian Industrial Co., Ltd, China). Standard samples were diluted to five concentrations: 1.5, 3, 6, 12 and 24 $\mathrm{ng}\;L^{-1}$ . Then, 1.8 mL of phosphate balanced solution (Biosharp, China) was added to 0.2 g of the sample at pH 7.2–7.4 and homogenised. The supernatant was centrifuged to obtain a sample. The concentrated washing solution was then diluted with distilled water for subsequent use. Samples were added to an ELISA plate, and blank, standard and sample pores were prepared for testing. We sealed the plate with a sealing film and held it in a warm bath at 37 °C for 30 min. The plate was then washed with detergent, and enzyme-labelled reagents were added, except in the case of the blank holes. The plate was then sealed with sealing film and placed in a warm bath at 37 °C for 30 min. After washing with detergent, developers A and B were added for 10 min to avoid light and to develop the colour. Termination solution was added to each well to terminate the reaction. Zeroing with blank holes and measurement of the wavelengths of each hole were performed at 450 nm. The concentration of each sample was calculated using linear regression based on the concentration of the standard substance and the measured photometric values.

#### 2.5.2 Myoglobin content

Myoglobin in meat was measured using a microplate reader. Then 0.2 g of the sample was added to 1 mL of 0.04 $\mathrm{mol}\;L^{-1}$ ( pH=6.8 ) phosphate buffer to homogenise in the homogenate. The sample was placed in ice water, was left to stand for 1 h and was then centrifuged (4 °C , 3500 $r\;\min^{-1}$ ) for 20 min. The supernatant was filtered and placed in a 96-well plate. A full-wavelength ELISA was used to determine the absorbance of the sample at wavelengths of 525, 545, 565 and 572 nm and was used to calculate the content as follows:

4 $$\begin{array}{rl} \text{Myoglobin} & \;(µ\mathrm{mol}\;L^{-1})=\\ & (-0.166A_{572\;\mathrm{nm}}+0.086A_{565\;\mathrm{nm}}\\ & +0.088A_{545\;\mathrm{nm}}+0.099A_{525\;\mathrm{nm}})\times 1000. \end{array}$$

### 2.6 Fatty acid composition and content

The composition and percentage content (%) of fatty acids were analysed through a gas chromatograph (Agilent, 7890A). To start with, 1.0 g of the sample was weighed, and then, after hydrolysis, 95 % ethanol solution was added. Subsequently, we added a mixture of ether and petroleum ether, mixing well, followed by lamination and desiccation to extract fat. For the quantification of fatty acid, the fatty acid methyl esters of each fat extract were prepared through water baths; centrifugation; and lamination by adding 2 % sodium hydroxide methanol solution, 14 % boron fluoride methanol solution and n-hexane solution. The fatty acids were analysed using a gas chromatograph (Agilent, 7890A) with split injection, a flame ionisation detector (FID) and a chromatographic column (CD-2560; 100m×0.25mm×0.2µm ). The samples were injected using a split ratio of 1/10 at a constant flow rate of 0.5 $\mathrm{mL}\;\min^{-1}$ . The oven temperature was set to 130 °C for 5 min and then was increased to 240 °C at a rate of 4 ${}^{\circ}\;\min^{-1}$ and was finally maintained at that temperature for 30 min.

### 2.7 Nucleotide and volatile substances

#### 2.7.1 Nucleotide composition and content

The composition and content (%) of nucleotides were analysed using liquid chromatography (Agilent, 1200). Then 5.5 g of the sample was weighed, and 10 mL of 15 % perchloric acid was added for ultrasonic extraction and was centrifuged for 5 min at 4000 $r\;\min^{-1}$ . The supernatant was transferred to a centrifuge tube. After ultrasonic extraction, the residue was centrifuged, combined with the supernatant, diluted with ultrapure water, mixed evenly and analysed through the membrane.

#### 2.7.2 Volatile-substance composition and content

The composition and content (%) of volatile substances were analysed through gas chromatography (Thermo Scientific, TriPlus 500). Then 2.0 g of the sample was weighed, 2 mL of the saturated sodium chloride solution was added and mixed well, and the headspace was measured.

### 2.8 Statistical analysis

Data for slaughter performance and meat quality are presented as the mean. Data were analysed using SPSS 26.0. One-way analysis of variance (ANOVA) and Duncan's multiple-comparison method were used to inspect differences between groups, where p<0.05 was considered to be the statistical significance.

## 3 Results

### 3.1 Slaughter performance

The breed of meat rabbits had a prominent effect on slaughter performance (Table 2). The pre-slaughter, half-bore and full-bore weights of Hyla rabbits were the highest, followed by those of the Laiwu black and the Minxinan black rabbits. The half-bore and full-bore weights of Minxinan black rabbits were significantly lower than those of the other groups ( p<0.01 ).

**Table 2:** Slaughter performance of 84 d old Laiwu black, Minxinan black and Hyla rabbits.

	Rabbit group			SEM	p value
	Laiwu black	Minxinan black	Hyla		
Pre-slaughter weight, g	2449.00 ${}^{b}$	1558.60 ${}^{c}$	3033.70 ${}^{a}$	118.458	<0.001
Half-bore weight, g	1378.27 ${}^{b}$	770.44 ${}^{c}$	1637.10 ${}^{a}$	69.312	<0.001
Full-bore weight, g	1255.10 ${}^{b}$	678.90 ${}^{c}$	1460.00 ${}^{a}$	63.220	<0.001
Half-bore rate, %	56.26 ${}^{a}$	49.98 ${}^{b}$	54.19 ${}^{a}$	0.842	0.004
Full-bore rate, %	51.23 ${}^{a}$	43.94 ${}^{b}$	48.37 ${}^{a}$	0.822	<0.001

Note that different superscripts in the same row indicate prominent differences ( p<0.05 ). SEM: standard error of the mean.

### 3.2 Meat quality and nutrient content

The $a^{*}$ values (Table 3) of Laiwu black and Minxinan black rabbits were significantly higher than those of Hyla rabbits ( p<0.01 ). The shearing force and drip loss (Table 3) of Laiwu black rabbits were significantly lower than those of the other groups ( p<0.05 ). The lipid and nicotinic acid contents (Table 4) in the meat of Laiwu black rabbits were significantly higher than those in the meat of the other groups ( p<0.01 ). The vitamin E content (Table 4) in the meat of Minxinan black rabbits was significantly lower than that in the meat of Hyla rabbits ( p<0.05 ). The protein content (Table 4) in the meat of Laiwu black and Minxinan black rabbits was significantly lower than that in the meat of Hyla rabbits ( p<0.01 ).

**Table 3:** Physical properties of meat from Laiwu black, Minxinan black and Hyla rabbits.

	Rabbit group			SEM	p value
	Laiwu black	Minxinan black	Hyla		
pH ${}_{45\;\min}$	6.67	6.72	6.66	0.042	0.807
pH ${}_{24\;h}$	5.79	5.63	5.71	0.044	0.321
$L_{45\;\min}^{*}$	42.20	41.85	43.10	0.474	0.556
$a_{45\;\min}^{*}$	5.47 ${}^{a}$	5.65 ${}^{a}$	4.46 ${}^{b}$	0.155	0.001
$b_{45\;\min}^{*}$	5.90	6.01	5.57	0.124	0.334
Shearing force, N	26.28 ${}^{b}$	30.97 ${}^{a}$	31.07 ${}^{a}$	0.781	0.011
Drip loss, %	1.72 ${}^{b}$	2.92 ${}^{a}$	2.58 ${}^{a}$	0.178	0.012
Water loss, %	39.07	41.22	38.36	0.548	0.081
Cooking loss, %	62.95	62.63	62.14	0.322	0.598

Note that different superscripts in the same row indicate prominent differences ( p<0.05 ). SEM: standard error of the mean.

**Table 4:** Nutrient content in the meat of Laiwu black, Minxinan black and Hyla rabbits.

	Rabbit group			SEM	p value
	Laiwu black	Minxinan black	Hyla		
Dry matter, %	28.69	30.01	30.60	0.401	0.136
Protein, %	19.98 ${}^{b}$	19.13 ${}^{b}$	21.33 ${}^{a}$	0.316	0.002
Lipid, %	2.58 ${}^{a}$	1.70 ${}^{b}$	1.50 ${}^{b}$	0.165	0.003
Ash, %	1.12	1.18	1.16	0.017	0.369
Nicotinic acid, $\mathrm{mg}\;{\mathrm{kg}}^{-1}$	44.86 ${}^{a}$	27.05 ${}^{b}$	25.11 ${}^{b}$	2.951	<0.001
Vitamin E, $\mathrm{mg}\;{\mathrm{kg}}^{-1}$	2.40 ${}^{\text{ab}}$	2.06 ${}^{b}$	2.53 ${}^{a}$	0.085	0.041

Note that different superscripts in the same row indicate prominent differences ( p<0.05 ). SEM: standard error of the mean.

**Figure 1:**
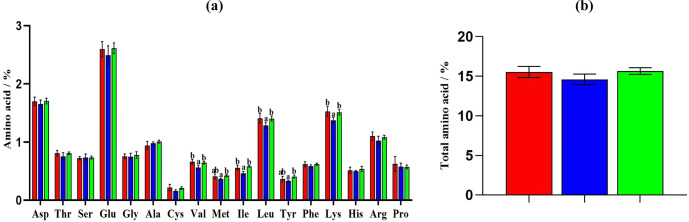
Comparison of amino acid contents of rabbit meat. **(a)** Contents of 17 amino acids. **(b)** Total amino acid contents of Laiwu black rabbits (red), Minxinan black rabbits (blue) and Hyla rabbits (green). Different superscripts in the three groups denote prominent differences ( p<0.05 ).

A total of 17 amino acids were determined in the rabbit meat (Fig. 1a). There was no significant difference in terms of the total amino acid content among the three rabbits ( p>0.05 , Fig. 1b). Among the 17 amino acids (Fig. 1a), there were prominent differences in 6 amino acids among the three groups, namely valine, methionine, isoleucine, leucine, tyrosine and lysine. The valine, isoleucine, leucine and lysine contents (Fig. 1a) in the meat of Laiwu black and Hyla rabbits were significantly higher than those in the meat of Minxinan black rabbits ( p<0.05 ). The methionine and tyrosine contents (Fig. 1a) in the meat of Hyla rabbits were significantly higher than those in Minxinan black rabbits ( p<0.05 ).

### 3.3 Melanin and myoglobin content

The breed of meat rabbit had a prominent impact on melanin content (Table 5). The melanin content in the meat of two local blank breeds was significantly higher than that in the meat of Hyla rabbits ( p<0.01 ). Moreover, the melanin content in the meat of Minxinan black rabbits was the highest, followed by that in Laiwu black rabbits ( p<0.01 ). The myoglobin content (Table 5) in the meat of Laiwu black and Minxinan black rabbits was significantly higher than that in the meat of Hyla rabbits ( p<0.01 ).

**Table 5:** Myoglobin and melanin content in the meat of Laiwu black, Minxinan black and Hyla rabbits.

	Rabbit group			SEM	p value
	Laiwu black	Minxinan black	Hyla		
Myoglobin, $µ\mathrm{mol}\;L^{-1}$	15.78 ${}^{a}$	15.87 ${}^{a}$	9.92 ${}^{b}$	0.858	0.002
Melanin, $\mathrm{ng}\;L^{-1}$	166.08 ${}^{b}$	215.51 ${}^{a}$	107.18 ${}^{c}$	13.876	<0.001

Note that different superscripts in the same row indicate prominent differences ( p<0.05 ). SEM: standard error of the mean.

### 3.4 Fatty acid composition and content

**Figure 2:**
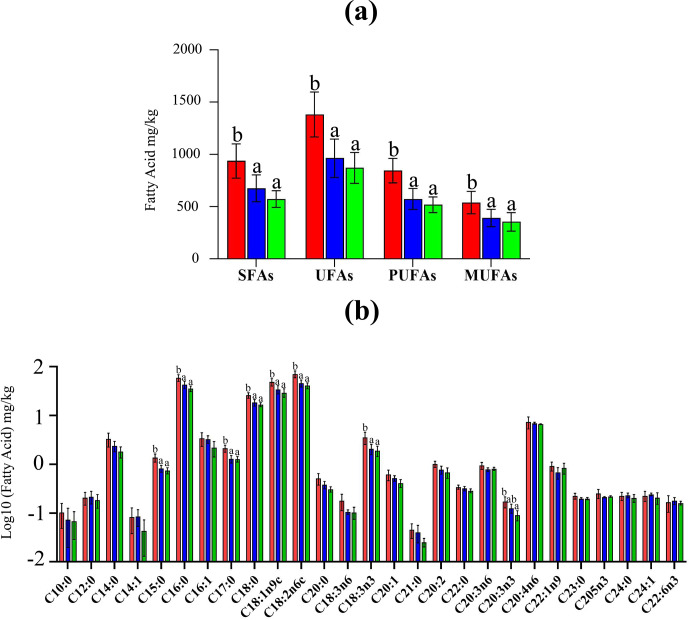
Comparison of fatty acid contents of rabbit meat. **(a)** Meat fatty acid types (SFAs – saturated fatty acids, UFAs – unsaturated fatty acids, PUFAs – polyunsaturated fatty acids, MUFAs – monounsaturated fatty acids) and **(b)** 27 kinds of fatty acid contents between Laiwu black rabbits (red), Minxinan black rabbits (blue) and Hyla rabbits (green). Different superscripts in the three groups denote prominent differences ( p<0.05 ).

The saturated fatty acid (SFA), unsaturated fatty acid (UFA), monounsaturated fatty acid (MUFA) and polyunsaturated fatty acid (PUFA) contents (Fig. 2a) in the meat of Laiwu black rabbits were significantly higher than those in the meat of the other groups ( p<0.05 ). In total, 27 types of individual fatty acids were identified (Fig. 2b), including 6 types of MUFAs and 9 types of PUFAs. A total of 8 types of fatty acid contents showed prominent differences out of this total of 27; specifically, 4 types of SFAs (pentadecanoic, palmitic, margaric, stearic acid), 1 type of MUFA (oleic) and 3 types of PUFAs (linoleic α -linolenic, eicosatrienoic) in the meat of Laiwu black rabbits were significantly higher than those in the meat of Hyla rabbits ( p<0.05 ). The contents of seven fatty acids other than eicosatrienoic showed prominent differences between Laiwu black rabbits and Minxinan rabbits, being significantly lower in the meat of Minxinan rabbits than in the meat of Laiwu black rabbits ( p<0.05 ). The contents of eight fatty acids in the meat of Minxinan black rabbits were not significantly different with those in Hyla rabbits ( p>0.05 ).

### 3.5 Nucleotide content and volatile-substance content

The uracil content (Table 6) in the meat of Laiwu black rabbits was significantly higher than that in the meat of the other groups ( p<0.05 ). In addition, the guanine and adenine contents (Table 6) in the meat of Minxinan black rabbits were significantly lower than those of the other groups ( p<0.01 ). The heptanal and octanal contents (Table 6) in the meat of Minxinan black rabbits were significantly higher than those of the other groups ( p<0.01 ). The caproic acid content (Table 6) in the meat of Laiwu black rabbits was significantly lower than that in the meat of Minxinan black rabbits ( p<0.05 ).

**Table 6:** Nucleotide and volatile-substance content in the meat of Laiwu black, Minxinan black and Hyla rabbits ( $µg\;{\mathrm{kg}}^{-1}$ ).

	Rabbit group			SEM	p Value
	Laiwu black	Minxinan black	Hyla		
Cytosine	5.71	4.95	4.35	0.247	0.062
Uracil	32.15 ${}^{a}$	23.37 ${}^{b}$	27.07 ${}^{b}$	1.362	0.011
Guanine	44.66 ${}^{a}$	37.22 ${}^{b}$	44.56 ${}^{a}$	1.229	0.003
Adenine	2023.47 ${}^{a}$	1558.77 ${}^{b}$	1924.95 ${}^{a}$	69.211	0.001
Hypoxanthine	163.62	176.58	166.87	3.632	0.348
Hexanal	261.93	477.08	300.53	52.149	0.210
Heptanal	15.15 ${}^{b}$	23.78 ${}^{a}$	18.38 ${}^{b}$	1.321	0.008
Octanal	2.25 ${}^{b}$	4.95 ${}^{a}$	3.28 ${}^{b}$	0.414	0.008
Nonanal	4.40	11.48	8.55	1.438	0.125
Caproic acid	491.00 ${}^{b}$	714.60 ${}^{a}$	551.88 ${}^{\text{ab}}$	40.346	0.045

Note that different superscripts in the same row indicate prominent differences ( p<0.05 ). SEM: standard error of the mean.

## 4 Discussion

Laiwu black and Minxinan black rabbits are genetic livestock resources in China; they have good meat quality, breeding efficiency and extensive adaptability; however, the growth velocity of Minxinan black rabbits is slow. Hyla rabbits are imported as a synthetic line with a high survival rate and a rapid growth rate. In this study, the pre-slaughter body weight, half-bore weight and full-bore weight of Hyla, Laiwu black and Minxinan black rabbits decreased sequentially, which is consistent with other reports on the growth and development rates of the three groups. Half-bore and full-bore rates are important indices for measuring the slaughter performance of meat rabbits and are affected by breed, nutrition and feeding system (Atsbha et al., 2021). In this study, the dressing percentage of Minxinan black rabbits was significantly lower than that of Laiwu black and Hyla rabbits, indicating that the slaughter performance of Laiwu black and Hyla rabbits is better under the same feeding and management conditions. Meat quality can be influenced by the growth rates of livestock and poultry (Hiscock et al., 2022). Therefore, it is of great significance to analyse the differences in meat quality between two local breeds and Hyla commercial rabbits.

Tenderness is an important indicator of meat quality. Meat tenderness was determined by the meat fibre content and characteristics of the intramuscular connective tissue. The shearing force is a numerical indicator of meat tenderness. The shearing force and drip loss of Laiwu black rabbits were significantly lower than those of Minxinan black and Hyla rabbits, and the water-holding capacity of the meat was negatively related to drip loss. The juiciness of meat is determined by its water-holding capacity and is positively correlated with meat tenderness (Bai et al., 2023). In summary, the meat tenderness of Laiwu black rabbits was better than that of Minxinan black and Hyla rabbits. According to Luo et al. (2018) and Long et al. (2022), respectively, the shearing force value of beef is approximately 60 N, and that of mutton is approximately 40 N, both of which are higher than that of rabbit meat. The results of this study indicate that rabbit meat is more tender than meat from other herbivorous animals such as cattle and sheep.

Meat colour is an important trait of meat quality, and meat myoglobin content is considered to be a prominent trait representing meat colour (Q. Liu et al., 2021). Myoglobin content in the meat of Laiwu black and Minxinan black rabbits was significantly higher than that in the meat of Hyla rabbits. Meat contains three primary forms of myoglobin: bright-red oxymyoglobin (OxyMb), purplish-red deoxymyoglobin (DeoMb) and brown metmyoglobin (MetMb). Higher $a^{*}$ values in meat are likely to be related to higher myoglobin contents (Wang et al., 2016). The Laiwu black and Minxinan black rabbits had higher $a^{*}$ values than the Hyla rabbits. These results indicate that the meat colour of Laiwu black and Minxinan black rabbits is superior to that of Hyla rabbits. In addition, the melanin content in the meat of Laiwu black and Minxinan black rabbits is significantly higher than that in the meat of Hyla rabbits. Melanin content is high in black livestock and poultry, which can enhance the darkness of meat colour (Kriangwanich et al., 2021). Otherwise, it has been confirmed that melanin serves many physiological functions, such as anti-oxidation, anti-ageing and anti-mutagenesis (Guo et al., 2023).

Meat is an important source of proteins, amino acids (AAs), fatty acids, nicotinic acid and vitamin E, which determine nutritional quality (Pereira and Vicente, 2013). The three branched-chain AAs, leucine (LEU), isoleucine (ILEU) and valine (VAL), in the meat of Laiwu black and Hyla rabbits were significantly higher than those of Minxinan black rabbits. There are essential AAs used by tissues as substrates for protein synthesis and fat deposition (Green et al., 2016). Lys can affect the metabolism of various enzymes and is catabolised as an energy source when sugar content is lower (Liao et al., 2015). The nicotinic acid and intramuscular fat contents in the meat of Laiwu black rabbits were significantly higher than those in the meat of Minxinan black and Hyla rabbits. Nicotinic acid is an important adipose regulator, and it can enhance the content of the intramuscular fat of Chinese crossbred finishing steers (B. Zhang et al., 2021), which also can reduce fat deposition in Ningxing pigs (Wang et al., 2022). However, its regulation of fat in rabbit meat is rarely reported, and the relationship between the content of nicotinic acid and the fat content of rabbit meat needs to be investigated.

The crude lipid, SFA, UFA, MUFA and PUFA contents in the meat of Laiwu black rabbits were significantly higher than those in the meat of Minxinan black and Hyla rabbits. SFAs and MUFAs are beneficial for intramuscular fat deposition (X. Zhang et al., 2022). In addition, fatty acids also play an important role in meat flavour (Hoa et al., 2021; Honrado et al., 2022). In the meat of Laiwu black rabbits, the contents of C18 fatty acids, which are essential for meat flavour, were significantly higher than those in Minxinan black and Hyla rabbits. Among C18 fatty acids, the regulation of the circadian rhythm of lipid metabolism by oleic acid can prevent and/or treat obesity and associated diseases; linoleic acid can decrease the risk for cardiovascular disease by regulating lipid metabolism (Martín-Reyes et al., 2023; Froyen and Burns-Whitmore, 2020). Meanwhile, oleic acid (C18:1) and linoleic acid (C18:2) can be degraded to aldehyde flavour substances and can produce a sweet, pleasant aroma; linolenic acids (C18:3) can produce fruity and buttery aromas (Bassam et al., 2022). The linoleic acid and α -linolenic acid contents in the meat of Laiwu black rabbits were significantly higher than those of Minxinan black and Hyla rabbits; these essential fatty acids are important in lipid metabolism and immune functioning (Murariu et al., 2023). ω -3 PUFAs can help in maintaining cardiac and vascular health and in preventing atherosclerosis (de Carvalho and Caramujo, 2018). Aldehyde content is often used as an indicator of freshness in meat (Frank et al., 2020). The heptanal and octanal contents in the meat of Minxinan black rabbits were significantly higher than those in Laiwu black and Hyla rabbits, and the caproic acid content in the meat of Minxinan black rabbits was significantly higher than that in Laiwu black rabbits. Heptanal, octanal, and caproic acid are volatile flavour compounds (Xie et al., 2016). Among them, heptanal and octanal can produce pleasant fragrances; caproic acid can combine with various aroma-active compounds to produce a unique aroma in cooked meat (Pavlidis et al., 2021). The uracil content in the meat of Laiwu black rabbits was significantly higher than that in the meat of Minxinan black rabbits and Hyla rabbits. Uracil is a bitter-tasting compound (L. Zhang et al., 2021), and its content was low compared with other kinds of flavour in the three rabbit meats; as such, the effect of uracil on taste can be ignored. The guanine and adenine contents in the meat of Laiwu black and Hyla rabbits were significantly higher than those in the meat of Minxinan black rabbits. Guanine and adenine belong to flavour nucleotides and can improve umami, sweetness and richness and can weaken astringency, bitterness and astringency (Ismail et al., 2020). In summary, the sweetness and aroma of Laiwu black rabbits are relatively prominent, and their role in regulating lipid metabolism and immune functioning is superior to those of the other two experimental rabbits. Minxinan black rabbits are more prominent in terms of freshness and aroma.

## 5 Conclusion

Laiwu black and Minxinan black rabbits are Chinese local black meat rabbits. Although Laiwu black and Minxinan black rabbits are not dominant in terms of slaughter performance compared with Hyla rabbits, the content of LEU, ILEU, VAL, niacin and fatty acids in the meat of Laiwu black rabbits is relatively high, and their role in regulating lipid metabolism and immune functioning is better than in the case of Hyla rabbits. At the same time, the sweetness and aroma in the meat of Laiwu black rabbits are relatively prominent, and the freshness and aroma in the meat of Minxinan black rabbits are more prominent. In addition, the content of melanin in the meat of Minxinan black rabbits is relatively high. Based on these advantages, these two Chinese local black rabbits can meet consumers' pursuits for high-quality rabbit meat.

## Data Availability

Data will be made available upon reasonable request.
